# The early development of a combined micro- and full-field X-ray fluorescence analysis system using white X-rays at PLS-II

**DOI:** 10.1107/S1600577524011111

**Published:** 2025-01-01

**Authors:** Min Woo Kim, Kangwoo Ahn, Chang Hun Lee, Tae Joo Kim, JongYul Kim, Min-Su Han, Hyeong Uk Mo, Jina Kim, Hyun Wook Park, Ho Jae Kwak, Jong Hyun Kim

**Affiliations:** a Pohang Accelerator Laboratory (PAL), POSTECH, Pohang37673, Republic of Korea; b Neutron Science Division Korean Atomic Energy Research Institute Daejeon34057 Republic of Korea; c Korea National University of Heritage, Buyeo33115, Republic of Korea; d Department of Physics Kyungpook National University Daegu41566 Republic of Korea; e Department of Mechanical Engineering Pohang University of Science and Technology (POSTECH) Republic of Korea; Advanced Photon Source, USA

**Keywords:** full-field X-ray fluorescence, FF-XRF, micro-XRF, µ-XRF, synchrotron X-rays, single-exposure imaging, 2D X-ray detector

## Abstract

X-ray fluorescence (XRF) is widely used to analyze elemental distributions in samples. Micro-XRF (µ-XRF), the most basic conventional XRF technique, offers good spatial resolution through precise 2D scanning with a micrometre-sized X-ray source. Recently, synchrotron based XRF analysis platforms have achieved nano-XRF with highly focused X-rays using polycapillary optics or mirrors, leveraging the excellent coherence of synchrotron radiation. However, XRF techniques are hindered by long data acquisition times (exceeding several hours) due to their point-by-point scanning approach, impeding large-area elemental mapping. Full-field XRF (FF-XRF), developed in the 2010s and based on the high brilliance of synchrotron X-rays, enables significantly shorter (less than a few minutes) data acquisition times via single-exposure imaging using a 2D X-ray detector. Nevertheless, it is constrained by relatively low spatial resolution and sensitivity. Hence, a new XRF platform is required to accommodate resolution demands to cover diverse experimental purposes. In this study, we developed a preliminary model of a novel XRF system that combines micro- and full-field XRF setups to address these limitations. This system allows easy mode switching while maintaining the region of interest of the imaging system within a single apparatus, simply by rotating the sample to face either detector depending on research purposes. We anticipate that this new XRF system will be widely utilized in various research fields as the initial XRF setup at Pohang Light Source-II.

## Abstract 2

We present the development of a novel X-ray fluorescence (XRF) analysis system that integrates both micro-XRF and full-field XRF techniques at the 9D beamline of PLS-II.

## 1. Introduction

X-ray fluorescence (XRF) spectroscopy is a powerful analytical technique that provides detailed, accurate information about the elemental composition of samples. Environmental scientists use XRF to monitor pollution levels and detect contaminants in soil and water (Hu *et al.*, 2017 ▸; Li *et al.*, 2022 ▸). Archeologists rely on XRF to identify the elemental makeup of artifacts, revealing information about their origin, age and manufacturing technologies (Bottaini *et al.*, 2012 ▸; Brun *et al.*, 2016 ▸). In materials science, XRF is used to analyze the composition of metals, ceramics and other materials (Segura-Ruiz *et al.*, 2021 ▸; Gianoncelli *et al.*, 2020 ▸; Dawkins *et al.*, 2020 ▸). In biomedical research, XRF has helped to study the distributions of trace elements in biological tissues, providing key insights to understand various physiological and pathological processes (Chevrier *et al.*, 2022 ▸; Porfido *et al.*, 2023 ▸).

XRF can be categorized into two main types based on the data acquisition method: micro-XRF (µ-XRF) and full-field XRF (FF-XRF) (Romano *et al.*, 2014 ▸; Fittschen *et al.*, 2015 ▸; Xiong *et al.*, 2020 ▸). The most commonly used technique, µ-XRF, provides high spatial resolution on the scale of a few micrometres, enabling detailed analysis of small features and interfaces in samples (Howard *et al.*, 2020 ▸; Böning *et al.*, 2007 ▸). In this system, a small X-ray beam is used to obtain elemental information from specific points in the sample by detecting emitted X-rays. These points are scanned in 2D to create 2D images containing elemental information. The X-ray beam size directly affects the pixel resolution of XRF images; thus, reducing beam size is crucial for improving spatial resolution. Recently, in synchrotron X-ray facilities, nano-XRF has been achieved with highly focused X-rays using polycapillary optics or mirrors, leveraging the excellent coherence of synchrotron X-rays (Matsuyama *et al.*, 2020 ▸; Byrnes *et al.*, 2023 ▸; Vigani *et al.*, 2018 ▸). Nevertheless, owing to the scanning process, these XRF systems inevitably have poor temporal resolution, despite offering better spatial resolution than FF-XRF systems.

In comparison, FF-XRF systems developed in the 2010s and based on the high brilliance of synchrotron X-rays and 2D X-ray detectors, offer rapid elemental mapping over large sample areas (even larger than a few square millimetres), typically ranging from hundreds of micrometres to millimetres within minutes (De Samber *et al.*, 2019 ▸; Wilson *et al.*, 2016 ▸). These systems use an X-ray beam larger than the field of view (FOV) to induce areal characteristic X-ray emissions from regions of interest (ROIs) on the sample. FF-XRF uses a 2D detector to capture a 2D image during a single exposure. Because the 2D image is obtained with a very short exposure compared with µ-XRF, highly bright X-rays (*i.e.* synchrotron radiation) are essential for FF-XRF. Despite this advantage of very short data acquisition time, FF-XRF detectors have relatively large pixel sizes (low spatial resolution) ranging from tens to hundreds of micrometres (Veale *et al.*, 2018 ▸; Klysubun *et al.*, 2023 ▸), whereas µ-XRF achieves pixel sizes of a few micrometres (submicrometres in nano-XRF) (Matsuyama *et al.*, 2020 ▸; Byrnes *et al.*, 2023 ▸; Vigani *et al.*, 2018 ▸). Although the pixel size can be reduced by optically magnifying the ROI with a pinhole, the lower sensitivity of the detectors remains a limitation of FF-XRF (Fittschen *et al.*, 2015 ▸; Xiong *et al.*, 2020 ▸).

In recent years, there has been increasing demand for XRF systems capable of providing both high-spatial-resolution and large-area elemental mapping in a single apparatus (Silva *et al.*, 2017 ▸). To address this demand, we have developed a novel XRF system that integrates FF-XRF and µ-XRF capabilities into a single platform at the 9D beamline of the Pohang Light Source-II (PLS-II) in South Korea. Specifically, two detectors for FF-XRF and µ-XRF are positioned symmetrically around the sample, allowing mode switching by rotating the sample to face either detector. Although FF-XRF and µ-XRF data were not acquired simultaneously, this integrated system offers wide experimental coverage by combining the speed of FF-XRF with the high spatial resolution of µ-XRF. For example, it could enable analysis of unknown samples composed of complex elements by scanning large surfaces with FF-XRF, followed by precise examination of smaller areas with µ-XRF to reveal detailed chemical composition. In this paper, we present the design, development and characterization of our new integrated XRF system at PLS-II. We also demonstrate its capabilities through elemental mapping of an ancient coin.

## 2. Experiments

### 2.1. Source characteristics

The experiments were conducted on the 9D beamline at PLS-II in South Korea. X-rays from the 9D beamline have a high flux of 4 × 10^12^ photons s^−1^ mrad^−2^ (0.1% bandwidth)^−1^ at 20 keV with a beam current of 400 mA (Kwak *et al.*, 2023 ▸). The beam divergence is 0.34 mrad vertically and 8 mrad horizontally. The source-to-sample distance (17 m) provides good spatial resolution and a large beam size of 5.8 mm vertically and 100 mm horizontally. The white-beam X-rays produced by the bending magnets in the 9D beamline have wide spectral characteristics with an excellent signal-to-noise ratio (SNR). This high-flux white X-ray beam provides high temporal resolution with a wide energy spectrum during XRF experiments.

### 2.2. Experimental setup of µ-XRF and FF-XRF

**Figure 1:**
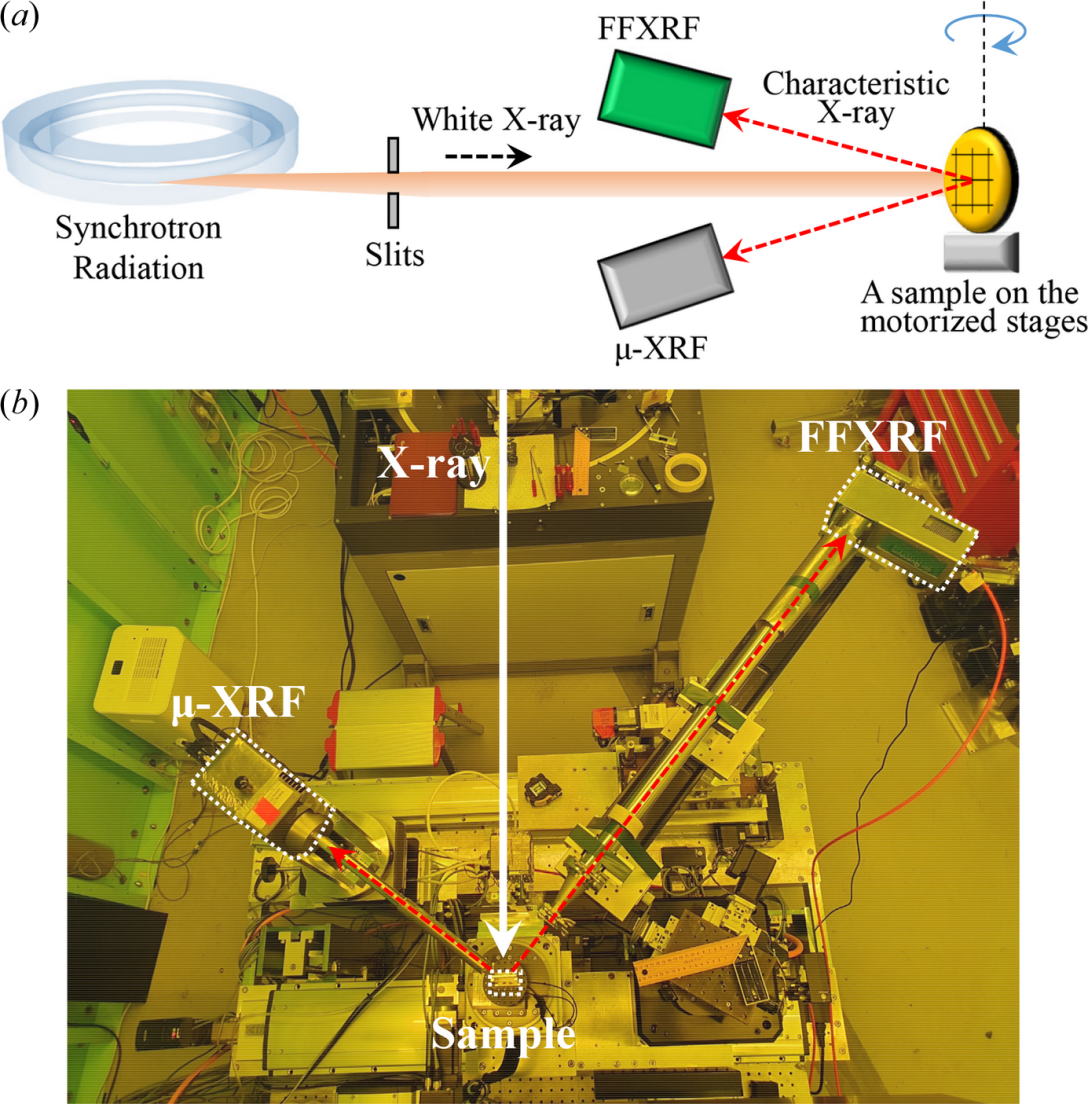
Combined µ-XRF and FF-XRF setup. (*a*) Schematic of the setup. The system has two detectors: a Vortex-EM for µ-XRF and a HEXITEC for FF-XRF. (*b*) Photograph of the setup. The white X-ray beam (white arrow) strikes the sample and induces characteristic X-rays (red arrows) that are detected by Vortex-EM and HEXITEC.

Fig. 1 ▸ illustrates the overall XRF system. As shown in Fig. 1 ▸(*a*), the white X-ray beam generated by the synchrotron radiation facility is restricted using slits to match the beam size to the FOV. This restriction minimizes X-ray damage to equipment and ozone gas production in the experimental hutch. When the white X-ray strikes a sample, characteristic X-rays are produced and detected by two detectors: one dedicated to µ-XRF (Vortex-EM, HITACHI, Japan) and the other to FF-XRF (HEXITEC, Quantum Detectors, UK). The sample stage comprises a rotation stage (RT150SX, LAB Motion Systems, Belgium) and three-axis linear nano-positioning stages (ECSxy5050 and ECSz3030, Attocube systems AG, Germany). The sample is placed on the motorized stages, allowing easy position control to align the beam between the sample and detectors. The two detectors are positioned opposite each other relative to the X-ray path to detect characteristic X-rays, as shown in Fig. 1 ▸(*b*).

**Figure 2:**
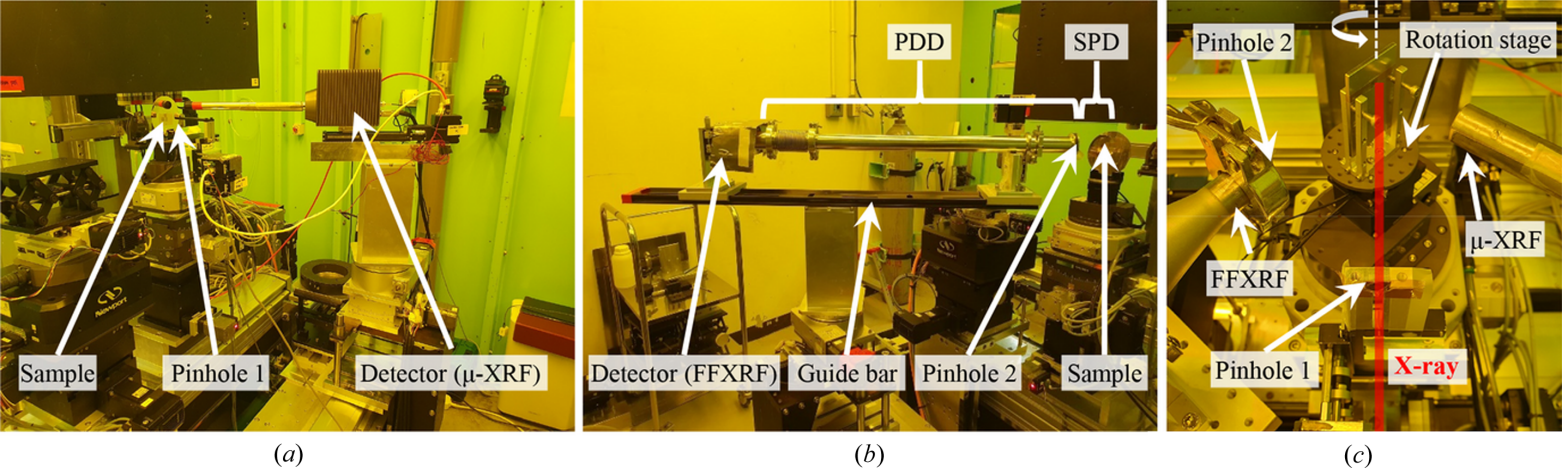
Photographs of the (*a*) µ-XRF and (*b*) FF-XRF setups developed here. (*c*) Photograph of a sample positioned on the motorized stages. Two pinholes are present: one for µ-XRF (pinhole 1) and one for FF-XRF (pinhole 2).

Fig. 2 ▸ presents photographs of the XRF system. The sample, pinhole 1 and the µ-XRF detector (Vortex-EM) are linearly aligned, as shown in Fig. 2 ▸(*a*). The Vortex-EM, a silicon drift X-ray detector, has 4096 channels for superior spectrum size. The X-ray beam diameter is reduced to approximately 5 µm after passing through a ∼5 µm-diameter pinhole in Pb. The beam size is an important factor because it directly affects the resolution of the µ-XRF system. The sample, pinhole 2 and FF-XRF detector (HEXITEC) are also linearly aligned, as shown in Fig. 2 ▸(*b*). HEXITEC is a 2D detector with a 1 mm CdTe sensor composed of 80 × 80 pixels (250 µm pixel^−1^) for the FF-XRF system. Unlike the µ-XRF system, the pinhole of the FF-XRF system magnifies the image, using the same principle as a pinhole camera. The magnification (*M*) is determined by the ratio of the pinhole-to-detector distance (PDD) to the sample-to-pinhole distance (SPD) as follows (Romano *et al.*, 2014 ▸),
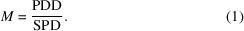
In this study, we tested magnifications of 10×, 20× and 25×. The detector is fixed 93 cm from the sample to facilitate magnification adjustment. The 50 µm-diameter pinhole for FF-XRF is positioned at 8 cm, 4.5 cm and 3.5 cm for 10×, 20× and 25×, respectively. The detector and pinhole positions are easily adjusted by sliding the components along a guide bar, as shown in Fig. 2 ▸(*b*). µ-XRF and FF-XRF are easily switched by rotating the sample stage toward the desired detector, as shown in Fig. 2 ▸(*c*). Pinhole 1, which reduces the size of the incident X-ray for µ-XRF, can be easily removed using a linear motorized stage to provide a large-area X-ray to the sample, making it suitable for FF-XRF. For the switch, the ROIs of the detectors are synchronized before the experiments.

## 3. Results

### 3.1. Image acquisition for µ-XRF and FF-XRF

µ-XRF data were collected using a 100 × 100 point scanning process with a 2 s pixel^−1^ dwell time (total approximately 6 h). The µ-XRF was operated using the *LabVIEW* software (National Instruments, USA). To visualize the data collected in 2D images, we utilized a self-developed *MATLAB* algorithm. In the algorithm, we could set a desired energy range for the visualization, and only pixel intensities within this range are displayed. Although the energy range was set for *K*α_1_ in this study since the demonstration samples were almost pure materials (>99%), other energy lines (*e.g.K*β_1_) could be used for precise visualization if needed. Energy calibration was conducted using test samples (Cu and Mo) prior to the experiments.

The FF-XRF images have 80 × 80 pixels, with a total exposure time of 30 s per image. The pinhole diameter is approximately 50 µm. The FF-XRF was operated using *LabVIEW* and the dedicated *HEXITEC* software (HEXITEC GigE data acquisition system) developed by the Science and Technology Facilities Council (STFC, UK) to visualize 2D images (Veale *et al.*, 2018 ▸).

### 3.2. Magnification-adjustable FF-XRF

**Figure 3:**
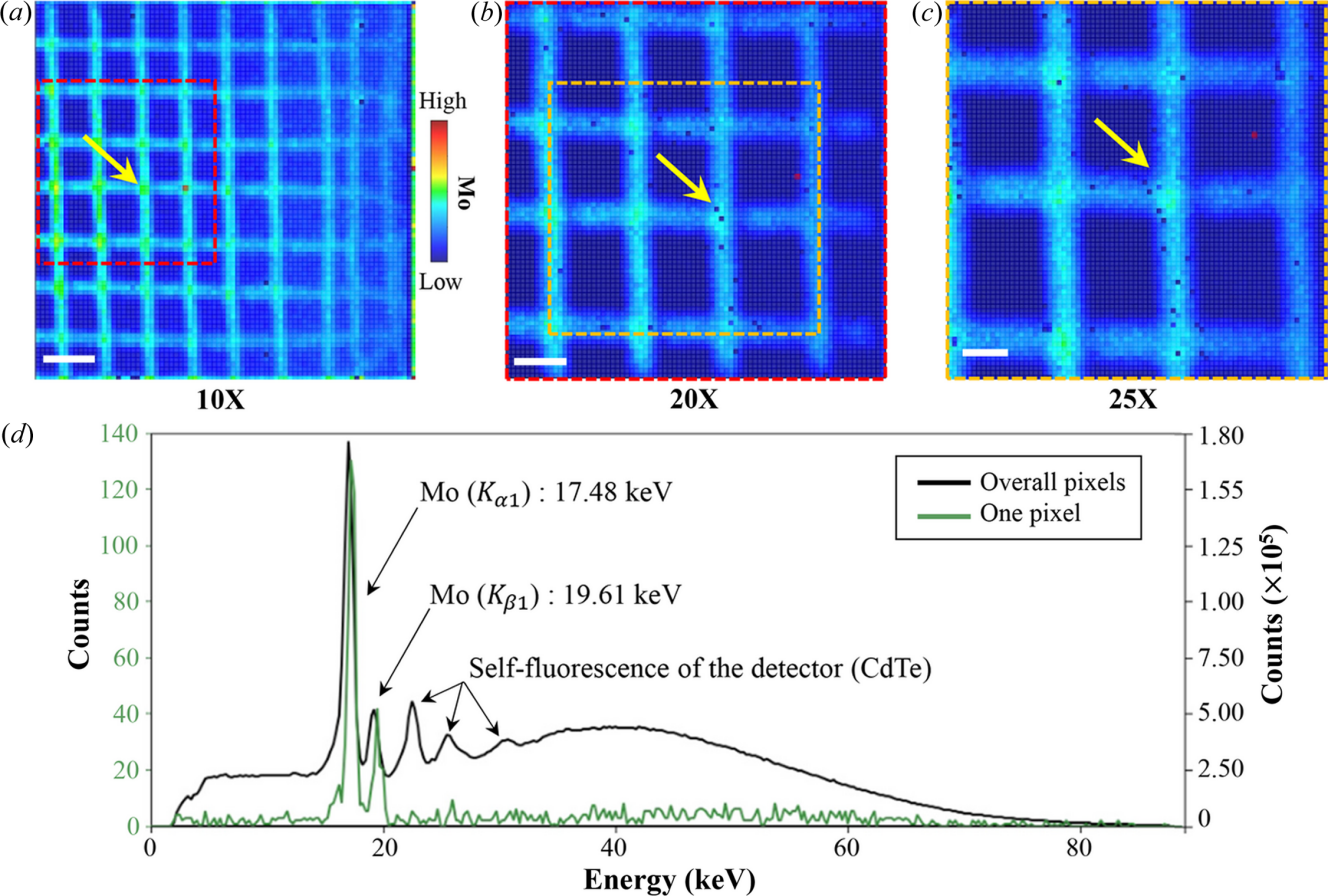
FF-XRF images of Mo mesh obtained at different magnifications. (*a*) Approximately 10× magnification: SPD = 8 cm, PDD = 85 cm; measured pixel size: 27.7 µm, scale bar = 300 µm. (*b*) Approximately 20× magnification: SPD = 4.5 cm, PDD = 88.5 cm; measured pixel size: 13.8 µm, scale bar = 150 µm. (*c*) Approximately 25× magnification: SPD = 3.5 cm, PDD = 89.5 cm; measured pixel size: 10.5 µm, scale bar = 100 µm. (*d*) XRF spectra for (*a*).

We used Mo mesh (The Nilaco Corporation, Japan) as a test sample while adjusting the magnification to 10×, 20× and 25×. The mesh consists of 50 µm-thick Mo wires with pitches of 200–250 µm. The XRF images at various magnifications exhibit high quality, as shown in Figs. 3 ▸(*a*)–3 ▸(*c*). The measured pixel sizes are 27.7 µm, 13.8 µm and 10.5 µm, respectively. Yellow arrows in the images indicate the same region of the mesh, suggesting that the magnification of the FF-XRF in the system can be adjusted while maintaining the ROI.

Fig. 3 ▸(*d*) shows the spectrum data measured from Fig. 3 ▸(*a*). The green line represents the spectrum corresponding to a pixel in the Mo wire region of the mesh. The black line represents the spectrum summed over all pixels in the image. The Mo *K* lines were observed at 17.48 keV (*K*α_1_) and 19.61 keV (*K*β_1_), as expected. Self-fluorescence of the detector was also detected from 23 keV to 31 keV, emitted by the *K* lines of Cd and Te. To minimize unexpected noise generated by scattered or emitted X-rays, the detector was shielded in Pb. However, because the source is a white beam, inevitable noise occurs from 3 keV to 90 keV, as described in Fig. 3 ▸(*d*), but the SNR is sufficient to detect characteristic X-rays emitted from pure material even with a short 30 s exposure.

### 3.3. Comparison of µ-XRF and FF-XRF

**Figure 4:**
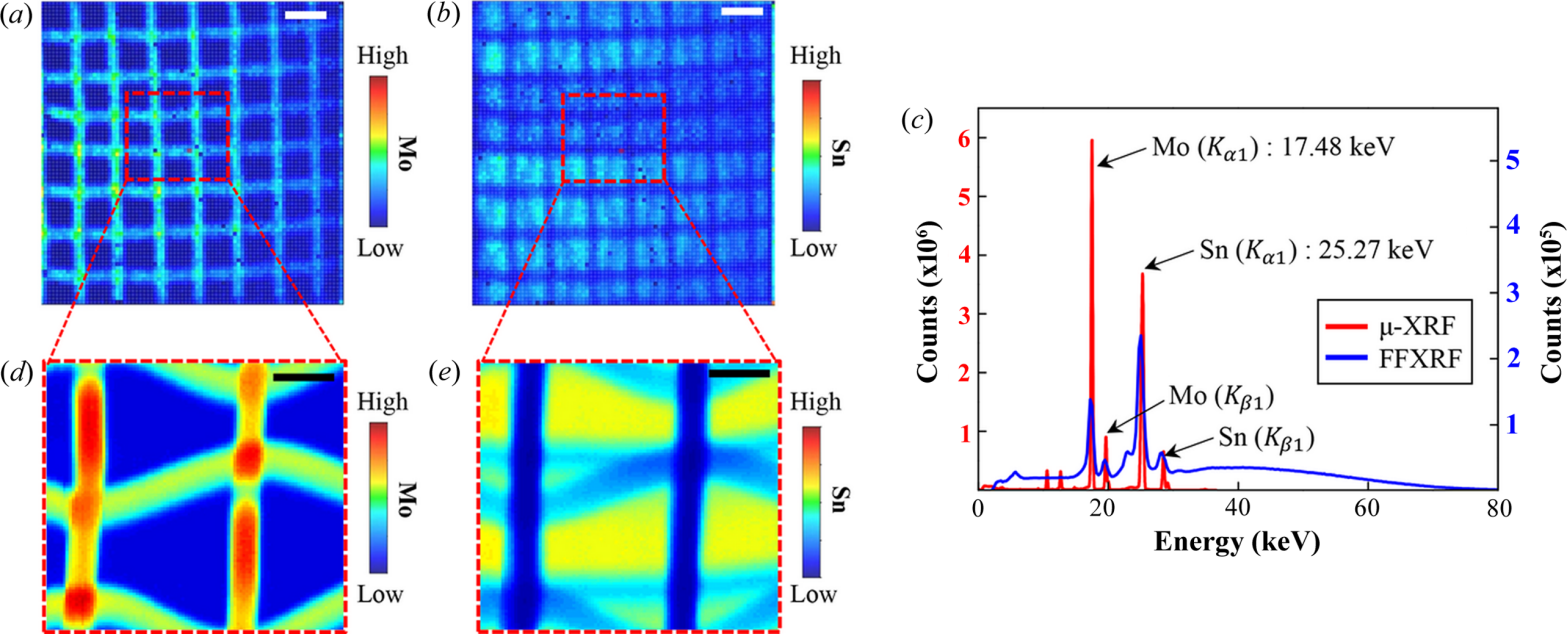
FF-XRF and µ-XRF XRF images of Mo mesh on an Sn sheet. (*a*) Mo in the FF-XRF (HEXITEC) image at 10× magnification, scale bar = 300 µm. (*b*) Sn in the same image. (*c*) XRF spectra of (*a*)–(*e*). The spectrum is obtained from overall pixels of each of the XRF images. (*d*) Mo in the µ-XRF (Vortex-EM) image in the red dashed box in (*a*), scale bar = 100 µm. (*e*) Sn in the µ-XRF image in the red dashed box in (*b*), scale bar = 100 µm.

**Figure 5:**
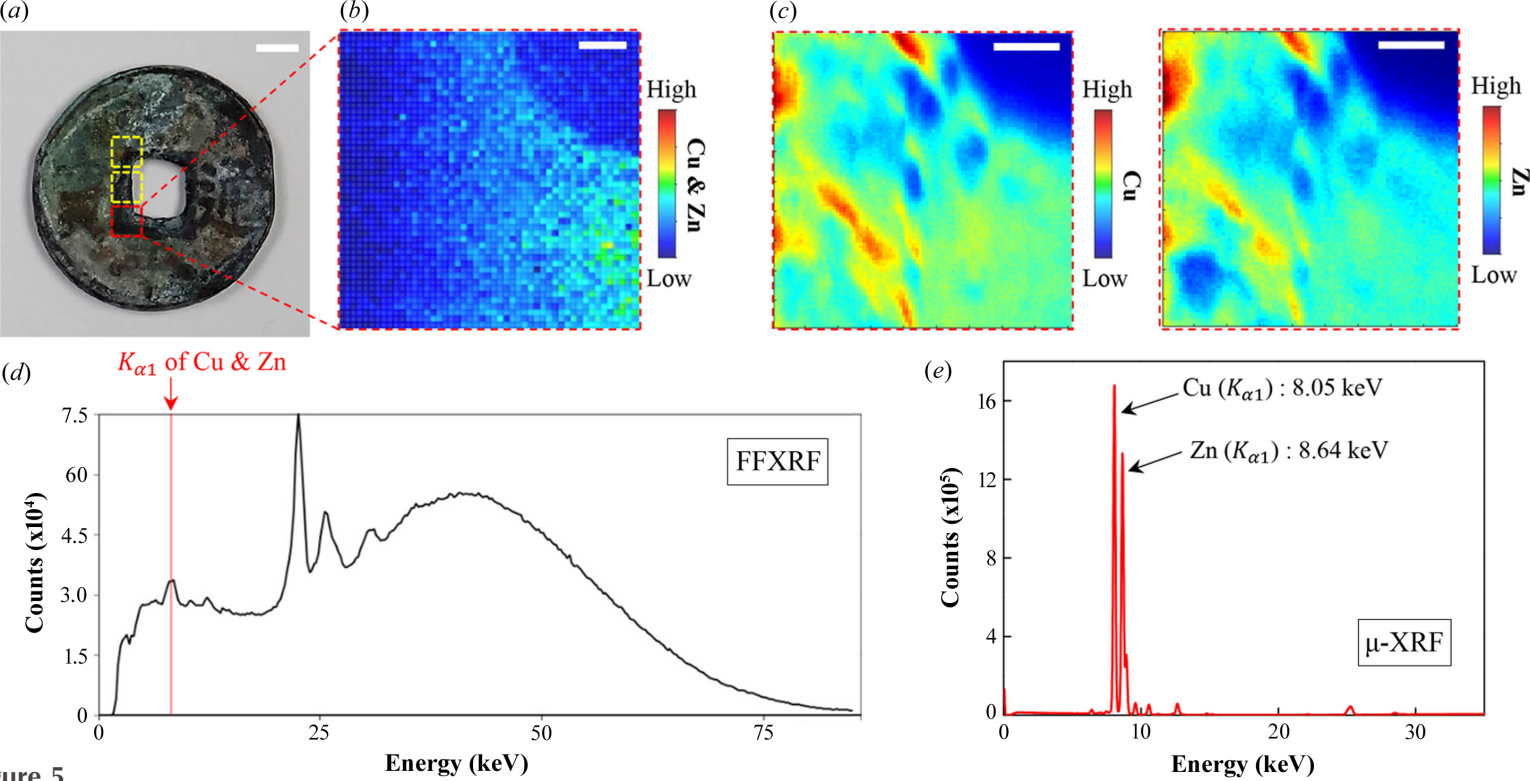
FF-XRF and µ-XRF images of an ancient Korean coin. (*a*) Optical microscopic image of the ancient coin, scale bar = 500 µm. Cu and Zn in the coin as assessed by (*b*) FF-XRF and (*c*) µ-XRF, scale bars = 100 µm. (*d*) and (*e*) XRF spectra of (*b*) and (*c*), respectively.

To compare the performances of FF-XRF and µ-XRF, Mo mesh (same specifications as in Fig. 3 ▸) on an Sn sheet (100 µm thickness) was used as a test sample. Figs. 4 ▸(*a*) and 4 ▸(*b*) show XRF images obtained by FF-XRF at 10× magnification. Mo and Sn in the sample were successfully visualized, as shown in Figs. 4 ▸(*a*) and 4 ▸(*b*), respectively. The Mo mesh blocked X-rays incident to and emitted from the Sn sheet, creating a mesh pattern shadow (low-Sn region) in Fig. 5 ▸(*b*). The *K* lines of Mo and Sn were detected at 17.48 keV (*K*α_1_) and 25.27 keV (*K*β_1_), as expected. Although some background noise is observed due to limited detector sensitivity, the *K* lines are distinguishable in the spectrum [blue line in Fig. 4 ▸(*c*)].

Figs. 4 ▸(*d*) and 4 ▸(*e*) show XRF images obtained by µ-XRF (100 × 100 pixels, 5 µm pixel^−1^). Mo and Sn in the red dashed box in Figs. 4 ▸(*a*) and 4 ▸(*b*) are well visualized, as shown in Figs. 4 ▸(*d*) and 4 ▸(*e*). The trapezoidal appearance of the mesh in the µ-XRF images is more noticeable in the high-resolution images, highlighting the importance of precise alignment between the detector and the sample. In Fig. 4 ▸(*c*), the Mo *K*α_1_ signal (17.48 keV) appears weaker in FF-XRF compared with µ-XRF. This discrepancy might be due to characteristics of the detectors. The detector used for FF-XRF in this study is a CdTe detector, which is specialized for high-energy detection. Moreover, our FF-XRF system currently lacks a vacuum environment along the beam path, which may reduce sensitivity in lower energy regions. The data acquisition time of µ-XRF (∼6 h) is much longer than that of FF-XRF. However, it offers better spatial resolution than FF-XRF images. Furthermore, the SNR in Figs. 4 ▸(*d*) and 4 ▸(*e*) is much higher than that of FF-XRF [red line in Fig. 4 ▸(*c*)].

### 3.4. Practical application example

Fig. 5 ▸ shows the XRF analysis results of an ancient coin from the Joseon Dynasty of Korea based on FF-XRF and µ-XRF imaging of the red box in Fig. 5 ▸(*a*). The coin is known to contain Fe, Ni, Cu, Zn, Pb and Se. The dominant elements by weight percentage are Cu (64.1%) and Zn (31.3%), as analyzed with a commercial XRF system. Figs. 5 ▸(*b*) and 5 ▸(*c*) show the FF-XRF and µ-XRF images, respectively. The FF-XRF data were measured with a 30 s exposure, whereas the µ-XRF data required about 6 h. Instead, the µ-XRF image exhibits higher quality than the FF-XRF image. Furthermore, as observed in Figs. 5 ▸(*d*) and 5 ▸(*e*), the spectra demonstrate that µ-XRF has a higher energy resolution than FF-XRF. Specifically, while Cu and Zn in the coin cannot be distinguished by FF-XRF [Fig. 5 ▸(*d*)], the elements are discernible with µ-XRF [Fig. 5 ▸(*e*)], despite their similar *K* lines (Cu, 8.05 keV; Zn, 8.64 keV). From these data, we estimated that the Cu and Zn concentrations were 49.5 ± 3.2% and 39.6 ± 1.5%, respectively, by averaging three regions for the red box and two yellow boxes in Fig. 5 ▸(*a*). The estimated composition differs slightly from the commercial XRF findings, possibly due to differences in the measured areas of the coin. In this system, we could rapidly explore ROIs with FF-XRF and perform precise measurements with µ-XRF. Because the advantages and disadvantages of µ-XRF and FF-XRF are complementary, their combination in a single system is useful for various applications.

## 4. Discussion and conclusions

In this study, we developed a combined µ-XRF and FF-XRF platform using white X-rays at PLS-II. This system addresses the limitations of traditional µ-XRF and FF-XRF by integrating the strengths of both techniques in a single platform, offering an easily switchable XRF system between high spatial resolution and rapid large-area elemental mapping capabilities for diverse experimental purposes.

The experimental results demonstrate the efficiency of the combined system in data acquisition and precise analysis. The µ-XRF setup, equipped with a Vortex-EM detector, provided detailed elemental maps with a spatial resolution of approximately 5 µm. The high-resolution images of an ancient coin reveal the ability of the system to identify and visualize small features with high precision. However, the data acquisition time for µ-XRF remains a key limitation, requiring hours for complete mapping due to the point-by-point scanning process. Conversely, the FF-XRF setup, using the HEXITEC detector, demonstrated rapid elemental mapping over large sample areas. Despite lower spatial resolution than μ-XRF, the FF-XRF system effectively visualized sample elemental composition even though it has low sensitivity, considering its brief data acquisition time (<30 s). Thus, FF-XRF can identify ROIs before detailed µ-XRF scanning. In previous studies (Alfeld *et al.*, 2011 ▸; Romano *et al.*, 2017 ▸), µ-XRF was performed using larger step sizes, known as macro-XRF, to roughly locate ROIs before precise scanning. This process required 10–30 min (depending on experimental conditions) but can be omitted using our combined XRF system. The adjustable magnification enhances the versatility of the FF-XRF setup, allowing adaptations to various experimental requirements.

Despite the convenience of our system, it has a few limitations. First, it cannot visualize µ-XRF and FF-XRF simultaneously due to their contradictory imaging principles. µ-XRF requires a small-point X-ray for better spatial resolution, whereas FF-XRF needs an area X-ray at least the size of the FOV to visualize a 2D image in one exposure. Thus, we designed the combined XRF setup to be easily switchable between experimental modes by rotating the sample stage and inserting (µ-XRF mode) or removing (FF-XRF mode) the pinhole in the X-ray path. Second, the HEXITEC detector for FF-XRF lacks sufficient resolution to analyze detailed elemental compositions of an ancient coin. This limitation is due to the CdTe detector (HEXITEC) performance, not a defect in the setup design. The system could be improved using a detector with better spatial and energy resolution than HEXITEC. Third, with a higher photon flux, shorter exposures could be used for FF-XRF. Improved temporal resolution would enable XRF to be widely used for observing dynamic phenomena. Fourth, the spatial resolution of µ-XRF was limited to 5 µm due to challenges in fabricating an extremely small pinhole in a lead sheet. Further X-ray focusing using optics such as Kirkpatrick–Baez mirrors or polycapillary optics could reduce the X-ray beam size to submicrometre levels and shorten th dwell time. These upgrades would provide much better spatial and energy resolution, broadening the research applications of the combined XRF system.

To our knowledge, no currently available XRF platform can perform both µ-XRF and FF-XRF to accommodate various demands regarding resolution and cover diverse experimental purposes in a single system by simply switching modes. By merging the high spatial resolution of µ-XRF with the rapid data acquisition capabilities of FF-XRF, the new system addresses the limitations of traditional XRF techniques and offers a versatile tool for comprehensive elemental analysis. Its successful application to various samples and potential for future enhancements make this system a valuable asset for researchers across diverse scientific fields.

## Data Availability

The datasets used and/or analyzed during the current study are available from the corresponding author on reasonable request.
